# Weighted composite time to event endpoints with recurrent events: comparison of three analytical approaches

**DOI:** 10.1186/s12874-022-01511-1

**Published:** 2022-02-05

**Authors:** Ann-Kathrin Ozga, Geraldine Rauch

**Affiliations:** 1 Institute of Medical Biometry and Epidemiology, University Medical Center Hamburg-Eppendorf, Martinistraße 52, Hamburg, 20246 Germany; 2 Charité - Universitätsmedizin Berlin, corporate member of Freie Universität Berlin, Humboldt-Universität zu Berlin, Institute of Biometry and Clinical Epidemiology, Charitéplatz 1, Berlin, 10117 Germany

**Keywords:** Composite endpoint, Time to event, Recurrent events, Relevance weights

## Abstract

**Background:**

In clinical trials the study interest often lies in the comparison of a treatment to a control regarding a time to event endpoint. A composite endpoint allows to consider several time to event endpoints at once. Usually, only the time to the first occurring event for a patient is thereby analyzed. However, an individual may experience more than one non-fatal event. Including all observed events in the analysis can increase the power and provides a more complete picture of the disease. Thus, analytical methods for recurrent events are required. A challenge is that the different event types belonging to the composite often are of different clinical relevance. In this case, weighting the event types according to their clinical relevance is an option. Different weight-based methods for composite time to event endpoints were proposed. So far, there exists no systematic comparison of these methods.

**Methods:**

Within this work we provide a systematic comparison of three methods proposed for weighted composite endpoints in a recurrent event setting combining non-fatal and fatal events of different clinical relevance. We consider an extension of an approach proposed by Wei and Lachin, an approach by Rauch et al., and an approach by Bakal et al.. Comparison is done based on a simulation study and based on a clinical study example.

**Results:**

For all three approaches closed formula test statistics are available. The Wei-Lachin approach and the approach by Rauch et al. show similar results in mean squared error. For the approach by Wei and Lachin confidence intervals are provided. The approach by Bakal et al. is not related to a quantifiable estimand. The relevance weights of the different approaches work on different level, i.e. either on cause-specific hazard ratios or on event count.

**Conclusion:**

The provided comparison and simulations can help to guide applied researchers to choose an adequate method for the analysis of composite endpoints combining (recurrent) events of different clinical relevance. The approach by Wei and Lachin and Rauch et al. can be recommended in scenarios where the composite effect is time-independent. The approach by Bakal et al. should be applied carefully.

**Supplementary Information:**

The online version contains supplementary material available at (10.1186/s12874-022-01511-1).

## Background

The focus of many cardiovascular or oncologic trials lies in the comparison of a treatment to a control intervention with regard to a time to event endpoint like time to myocardial infarction, time to stroke, time to relapse, or time to death. Including only one of those event types can result in a large number of patients that need to be observed to gain an effect with sufficient power. To overcome this issue and decrease the required sample size, composite endpoints can be considered alternatively [1, 2]. Thereby, several events of interest can be combined and analyzed at once. Commonly, methods for analyzing the time to the first occurring event of an individual are applied, like the log-rank test or the Cox proportional hazards model [3]. Thus, it is neglected that an individual may experience more than one event, e.g. several myocardial infarctions or a myocardial infarction followed by death. Incorporating all events experienced by an individual increases the amount of information used for effect estimation and can further decrease the sample size due to the expected higher amount of events. It further provides a more complete picture of the disease process. Cox proportional hazards based models were introduced for the analysis of recurrent time to events like the Andersen-Gill model [4], the marginal model by Wei, Lin and Weissfeld [5], and conditional models by Prentice, Williams and Peterson [6]. In those models only one event type is considered and thus, when applied to a composite endpoint, it is implicitly assumed that a myocardial infarction has the same clinical relevance as death and the treatment effect is the same in both endpoints [7]. An alternative modelling approach for the combination of a recurrent event process and a fatal event process are so-called joint frailty models [8, 9]. Thereby, a correlation between events can be modelled and two effects are estimated, one for each event type. Although this seems to be an appealing approach, results are more difficult to interpret because they are conditioned on the so-called frailty parameter and a single all-cause effect is not provided. Such an all-cause effect should be able to ease the interpretation if the components are events of different clinical relevance. Weighted effect measures were proposed to consider the clinical relevance of the combined event types [10–13]. The common idea of these approaches is that a relevance weight is assigned to each event type with the aim to make the comparison between different events more fair. However, most of these weighted approaches were only described for the time to first event endpoint analysis.

Rauch et al. recently introduced the weighted all-cause hazard ratio where pre-defined relevance weights are multiplied to the cause-specific hazards [14, 15]. A corresponding closed formula test statistic was also provided [15]. Although the method was described for a time to first event analysis it can be easily extended to the situation of a time to recurrent event analysis as it is shown in the present work. Other weighting approaches for the analysis of a composite endpoint combining a recurrent non-fatal event with other fatal or non-fatal events were proposed: Bakal et al. proposed a weighted non-parametric approach [16, 17] and Wei and Lachin described a multivariate approach [18, 19] which is extended to recurrent events in this work. So far, the performance of these three methods in different clinical data scenarios was not analyzed and compared systematically. This would help to better understand the properties of the different approaches and to gain recommendations for or against their application. Therefore, in this work we provide a systematic comparison of the three methods (approach by Wei and Lachin, approach by Rauch et al., approach by Bakal et al.) with the help of a Monte-Carlo simulation study.

## Methods

We consider a two-arm clinical study with an intervention (*I*) and a control (*C*), where the primary endpoint is a composite time to event endpoint combining two event types. Throughout this work, it is assumed that there is one fatal event “death” (*D*) and the other non-fatal event is “myocardial infarction” (*M*). The non-fatal event might occur more than once per individual. An individual might also experience no event in the observational period. We consider classical continuous time to event data which are right censored. Although, we illustrate the approaches based only on two different event types, they can easily be applied to scenarios with e.g. more than one non-fatal event.

The total number of individuals *n* are randomized in a 1:1 allocation to the two groups. We consider a one-sided test problem, where the null hypothesis states that the control is better or equal to the intervention and the alternative states that the intervention group is superior. The test hypothesis are fomulated in terms of the underlying estimand for the specific model as specified below. Only for the approach by Bakal et al. there is no formal estimand and therefore no formal null hypothesis can be formulated.

### Formulation of the test problem and the estimand

In the following, the underlying test problems and the corresponding estimands will be formulated for the three weighted approaches under comparison. The test hypotheses are similar across methods, however it is important to highlight the differences in the underlying modelling approaches (see also Table 1).

**Table 1:** Comparison of analysis methods

	Wei-Lachin	Rauch	Bakal
Model assumptions	∙Stratified approach, i.e.	∙Stratified approach, i.e	Not applicable because no
	all 1st events in 1st stratum,	all 1st events in 1st stratum,	underlying model is specified.
	all 2nd events in 2nd stratum,	all 2nd events in second stratum,	
	and so on. I.e. individuals are at	and so on. I.e. individuals are at	
	risk for a subsequent event	riskrisk for a subsequent event	
	only if a previous event has occurred.	only if a previous event has occurred.	
Strong assumption	∙ Proportional hazards are	∙ Proportional hazards are	
	assumed within strata	assumed within strata	
	and event types.	and event types.	
	→ Equal cause-specific	→ Equal cause-specific baseline	
	baseline hazards.	hazards (or specific underlying	
		event distribution)	
	→ Baseline hazards can be	→ Baseline hazards can be	
	strata-specific, i.e. risk for	strata-specific, i.e. risk for	
	subsequent events	subsequent events	
	is allowed to change.	is allowed to change.	
Estimation assumptions	No difference to	Cause-specific hazards	∙ No difference between strata, i.e.
	model assumptions.	are different.	no risk change for subsequent event.
			∙ Individuals are at risk as long as
			they are under observation
			but their contribution to the
			event number and number at risk
			changes for subsequent events.
Weights	∙ pre-specified	∙ pre-specified	∙ pre-specified
	∙ non-negative	∙ non-negative	∙ non-negative
	∙ relative weights	∙ weights based on	∙ relative weights
		clinical relevance	
	∙ sum up to 1	∙ proposed highest weight of 1	∙ highest weight of 1 (for 1 type)
		but could be higher	
	∙ works multiplicatly on the	∙ works multiplicatly on the	∙ works accumulatively multiplicative
	logarithmized cause-specific	cause-specific hazards (event counts)	on the event count
	hazard ratios		
Test statistic	multivariate procedure	stratified weight based	modified log-rank test
	(semi-parametric)	log-rank test	(not stratified)
Effect estimator	$\checkmark $	$\checkmark $	x
Confidence interval for effect	$\checkmark $	only bootstrap	x
Interpretation	∙ Weighted cause-specific	∙ Weighted cause-specific	Weighted individual score
	logarithmic hazard ratios.	hazards work on	for event count and risk set.
	Thus influence of event counts is not	the event counts and hence is	
	directely incoporated, i.e. a higher	also satisfying in terms of variablity	
	cause-specific logarithmic hazard ratio	for a low event number.	
	has a higher influence on	Thus the composite effect is	
	the composite effect, which	determined by the distribution of	
	results in a higher	the clinically more relevant event.	
	variability when the estimation		
	is based on a low event number.		
	∙ weighted composite hazard ratio based	∙ weighted composite effect based on	
	on weighted cause-specific	weighted cause-specific hazards	
	logarthimic hazard ratios		

#### Approach by Wei and Lachin

In the works by Wei and Lachin [19] and Lachin et al. [18] only the time to the first event is considered. However, the approach can be easily extended to recurrent events by defining the hazard functions as stratified hazards, where the strata *j*=1,...,*J* define the subgroup of all first, second, third events etc.. The stratified hazards read as

1 $$\begin{array}{@{}rcl@{}} \lambda_{D,j}(t)&=&\lambda_{D,0j}(t)\cdot exp(\beta_{D}\cdot X)  \end{array} $$

2 $$\begin{array}{@{}rcl@{}} \lambda_{M,j}(t)&=&\lambda_{M,0j}(t)\cdot exp(\beta_{I}\cdot X)  \end{array} $$

where *X* is the group indicator and *X*=1 refers to the intervention group. This model implies that the cause-specific baseline hazards (*λ*_*D*,*j*0_(*t*),*λ*_*M*,*j*0_(*t*)) are strata-specific, i.e. hazards can change for subsequent events, but the cause-specific effect (*exp*(*β*_*D*_),*exp*(*β*_*M*_)) remain the same over all strata. This model moreover suggests proportional hazards for both event types within the strata.

Wei and Lachin [19] then define a so-called “weighted hazard ratio” as

3 $$\begin{array}{@{}rcl@{}} &&\theta^{L}:=exp(w^{L}_{D}\beta_{D}+w^{L}_{M}\beta_{M}),\\ &&w^{L}_{D}+w^{L}_{M}=1, \ w^{L}_{D}, w^{L}_{M}>0, \end{array} $$

where the index *L* denotes the Wei-Lachin weighting approach and $w^{L}_{D}$ and $w^{L}_{M}$ are the pre-specified relevance weights which are described to reflect the “relative importance or severity” [18]. The weights are working on the logarithmized cause-specific hazard ratios, but not directly on the hazard function. This implies, that the influence of the weight is independent from the underlying number of events, and as a consequence a high weight has a large impact even if the corresponding cause-specific hazard ratio is estimated based on a low number of events. The corresponding hypotheses are then formulated as follows:

4 $$\begin{array}{@{}rcl@{}} H_{0}:\theta^{L} \geq 1 \quad \text{versus} \quad H_{1}: \theta^{L} <1.  \end{array} $$

To test the above null hypothesis (4) the following test statistic was proposed [18]:

5 $$ T^{L}=\frac{w^{L}_{D}\hat\beta_{D} +w^{L}_{M}\hat\beta_{M}}{\sqrt{(w^{L}_{D})^{2}\hat\sigma^{2}_{D}+2 w^{L}_{D} w^{L}_{M}\hat\sigma_{D,M}+(w^{L}_{M})^{2}\hat\sigma^{2}_{M}}}  $$

where the estimators for the cause-specific logarithmic effects $\hat \beta _{D}$ and $\hat \beta _{M}$ can be obtained by using a stratified Cox-model for each cause. The corresponding variance estimators of *β*_*D*_ and *β*_*M*_ are denoted by $\hat \sigma ^{2}_{D}$ and $\hat \sigma ^{2}_{M}$, respectively and the covariance estimator of *β*_*D*_ and *β*_*M*_ as $\hat \sigma _{D,M}$. Lachin and Bebu [18] show in their supplement how $\hat \sigma ^{2}_{D}, \hat \sigma ^{2}_{M}$, and $\hat \sigma _{D,M}$ can be calculated. Further the function mmm in the R package multicomp also provides these values [20–22]. The test statistic *T*^*L*^ is asymptotically standard normally distributed under the null hypothesis. Thus, the null hypothesis is rejected if *T*^*L*^≤−*z*_1−*α*_, where *z*_1−*α*_ is the (1−*α*)-quantile of the standard normal distribution and *α* is the one-sided significance level.

By means of the estimators for the cause-specific logarithmic effects and their variances, the estimated weighted hazard ratio is given as:

6 $$\begin{array}{@{}rcl@{}} \hat\theta^{L}&:=&exp\left(w^{L}_{D}\hat\beta_{D} + w^{L}_{M}\hat\beta_{M}\right).  \end{array} $$

The corresponding (1−2·*α*)-confidence interval is given as:

7 $$ {}\begin{aligned} exp\left(log(\hat\theta^{L})\mp z_{1-\alpha}\cdot \sqrt{(w^{L}_{D})^{2}\hat\sigma^{2}_{D}+2 w_{D}^{L} w^{L}_{M}\hat\sigma_{D,M}+ (w^{L}_{M})^{2}\hat\sigma^{2}_{M}} \right). \end{aligned}  $$

#### Approach by Rauch et al.

Rauch et al. [14] recently described the so-called “weighted all-cause hazard ratio” for a composite time to first event endpoint which we here extend to recurrent time to event analysis. A non-parametric estimator for this approach was already described [15] and is now extended within this work to allow multiple events per patient. As before for the Wei and Lachin approach, the stratified cause-specific hazards given in (1) and (2) are considered. Thereby, it is assumed that if e.g. death occurs as a second event this event belongs to the second stratum.

The newly adapted definition by Rauch et al. [14] for the “weighted all-cause hazard ratio” is given as

8 $$\begin{array}{*{20}l} &\theta^{R}:=\frac{1}{J}\sum\limits_{j=1}^{J}{\frac{w_{D}^{R}\lambda_{D,j}^{I}(t)+w_{M}^{R}\lambda_{M,j}^{I}(t)}{w_{D}^{R}\lambda_{D,j}^{C}(t)+w_{M}^{R}\lambda_{M,j}^{C}(t)}},  \end{array} $$

9 $$\begin{array}{*{20}l} &w^{R}_{D}, w^{R}_{M}\geq 0, \end{array} $$

where the index *R* denotes the weighting approach by Rauch et al. and $w^{R}_{D}$ and $w^{R}_{M}$ are the pre-specified relevance weights. Note that in contrast to the Wei and Lachin approach the weights are not forced to sum-up to 1 since they are implemented in the numerator and the denominator. The weights are working on the hazard functions and not on the hazard ratios. As the hazard function estimator depends on the number of observed events, a high weight can still have a low impact if the underlying event rate is small. This is a fundamental difference to the approach of Wei and Lachin. Ozga and Rauch [15] proposed a guidance for the choice of weights where a weight of 1 is assigned to the most clinical relevant event. For all other event types a weight ≤ 1 is assigned. The weighted all-cause hazard ratio can be interpreted as the weighted average of cause-specific hazards/hazard ratios. In contrast, the weighted hazard ratio by Wei and Lachin does not directly transfer to the common all-cause hazard ratio.

The weighted all-cause hazard ratio defines a simple extension of the common all-cause hazard ratio, i.e. the common all-cause hazard ratio is gained if all weights are equal to 1.

The corresponding hypotheses for the weighted all-cause hazard ratio can be formulated as follows:

10 $$\begin{array}{@{}rcl@{}} H_{0}:\theta^{R}\geq 1 \quad \text{versus} \quad H_{1}: \theta^{R} <1. \end{array} $$

To test the above null hypothesis (10), Ozga and Rauch [15] proposed a (stratified) weight-based log-rank test statistic *T*^*R*^. The test statistic formula is given in the Additional File.

The test statistic *T*^*R*^ is approximately standard normal distributed. Thus, the null hypothesis is rejected if *T*^*R*^≤−*z*_1−*α*_, where *z*_1−*α*_ is the (1−*α*)-quantile of the standard normal distribution and *α* is the one-sided significance level.

Ozga and Rauch [15] described a non-parametric estimator for the weighted all-cause hazard ratio. The idea of the non-parametric estimator is to replace the hazard functions in (8) by the cumulative hazard functions, which results in the same estimator under the assumptions of equal baseline-hazards for the different event types:

11 $$\begin{array}{@{}rcl@{}} \hat{\theta}^{R}:=\frac{1}{J}\sum\limits_{j=1}^{J}{\frac{w^{R}_{D}\cdot \hat\Lambda_{D,j}^{I}(t) + w^{R}_{M}\cdot \hat\Lambda_{M,j}^{I}(t)}{w^{R}_{D}\cdot \hat\Lambda_{D,j}^{C}(t) + w^{R}_{M}\cdot \hat\Lambda_{M,j}^{C}(t)}}  \end{array} $$

where $\hat \Lambda _{D,j}^{I}(t), \hat \Lambda _{M,j}^{I}(t), \hat \Lambda _{D,j}^{C}(t)$, and $\hat \Lambda _{C,j}^{M}(t)$ are the cause-, group, and strata-specific Nelson-Aalen estimators for the cumulative hazards at time *t*. This non-parametric estimator was recently shown to be robust under deviations from the equal baseline-hazards assumption [15].

Because a variance estimator cannot be derived for the weighted all-cause hazard ratio, confidence intervals can only be gained via bootstrap sampling.

#### Approach by Bakal et al.

The method described by Bakal et al. [16, 17] is a non-parametric weighted estimation approach for the survival probabilities, i.e. a weighted procedure for the Kaplan-Meier estimate. However, they do not define any underlying model and as a consequence the estimand is unspecified. By this, there naturally also is no effect estimator. The approach is based on so-called “weighted survival functions”, however the weighting scheme is only described on the estimation level. Therefore, the formulation of formal test hypotheses is not possible.

The weights proposed by Bakal et al. [16, 17] are denoted by $w^{B}_{M}$ and $w^{B}_{D}$ ∈[0,1] where for fatal events or the most relevant event a weight of 1 is assigned and for non-fatal events a weight < 1 is used. They are working recursively on the observed event counts where the recursion is with respect to all previous events for an individual. The other event types are then set in relation to this most relevant event type. This choice of the weights is similar to the approach of Rauch et al. [14].

The estimated weighted survival probabilities can be gained in a two-stage process (an example can be found in the Additional File).

Thereby for each individual *i*, *i*=1,..,*n*, a weight $w_{i}^{B}(t_{k})$ corresponding to the observed individual event at time *t*_*k*_ is assigned where *t*_*k*_ are the ordered (not strata-specific) distinct event times for *k*=0,..,*K*, where *K* ist the maximum number of events per individual and *t*_0_=0. In our scenario $w_{i}^{B}(\cdot)$ can either be $w^{B}_{M}(\cdot)$ or $w^{B}_{D}(\cdot)$. All observations per individual are included with the respective weight.

Using this, the first step is to assign an individual score for each patient at all event time points. This score is used for calculating the net impact with which the individual events are included in the estimation of the weighted survival probability. The weighted survival probability thereby depends on the weighted event count and on a weighted number at risk. The idea is that instead of considering an event as either present or not, in the approach by Bakal et al. a patient can experience a partial event counting less than a full event which, as a consequence, reduces the risk set by an amount lower than 1.

Each individual starts with a score of 1, i.e. the individual is fully at risk for an event. This score is subsequently reduced as follows: if the patient experienced a non-fatal event (weight smaller than 1) the patient remains partly at risk and if a fatal event was observed (weight equal to 1) the patient is removed from the risk set. Formally, this reads as:

**1.** Assign an individual score *s*_*i*_(·),*i*=1,...,*n*, for all observed event times *t*_*k*_,*k*=1,...,*K*:

12 $$\begin{array}{*{20}l} s_{i}(t_{k})&=s_{i}(t_{k-1})-\lbrack s_{i}(t_{k-1})\cdot w_{i}^{B}(t_{k})\rbrack, \end{array} $$

13 $$\begin{array}{*{20}l} s_{i}(t_{0})&=1. \end{array} $$

**2.** As a second step the weighted survival probabilities are calculated by replacing the event counts by the above defined scores.

For this we define the total number of weighted events at *t*_*k*_ as:

14 $$\begin{array}{@{}rcl@{}} e_{k}^{B}:=\sum\limits_{i=1}^{n} s_{i}(t_{k-1})-\sum\limits_{i=1}^{n} s_{i}(t_{k}). \end{array} $$

Further the total number of individuals at risk at *t*_*k*_ are defined as:

15 $$\begin{array}{@{}rcl@{}} n_{k}^{B}:=\sum\limits_{i=1}^{n} s_{i}(t_{k-1}). \end{array} $$

Note, individuals can be only partly at risk as long as they are still under observation, i.e. had no fatal event or were censored but had a non-fatal event.

Analogously, the group-specific number of weighted events and number of individuals at risk can be defined, denoted by an additional upper index *I* or *C*.

Using this, the survival probabilities can be calculated (recursive formula for Kaplan-Meier estimate):

16 $$\begin{array}{*{20}l} KM^{B}(t_{k})&=KM^{B}(t_{k-1})\cdot \left(1-\frac{e_{k}^{B}}{n_{k}^{B}}\right), \end{array} $$

17 $$\begin{array}{*{20}l} KM^{B}(t_{0})&=&1. \end{array} $$

For group-wise calculation of these weighted survival probabilities only the corresponding individuals and weights within the groups are used. As mentioned in the publication of Westerhout et al. [17] the common log-rank test can be used in a modified version to test the hypothesis whether these weighted survival probabilities for the groups are the same.

The test-statistic is given as follows:

18 $$\begin{array}{@{}rcl@{}} T^{B}=\frac{\sum_{k=1}^{K}\left(e_{k}^{B,I}-\frac{n_{k}^{B,I}\cdot e_{k}^{B}}{n_{k}^{B}}\right)} {\sqrt{\sum_{k=1}^{K}\frac{n_{k}^{B,I}\cdot n_{k}^{B,C}\cdot(n_{k}^{B}-e_{k}^{B})\cdot e_{k}^{B}}{(n_{k}^{B})^{2}\cdot(n_{k}^{B}-1)}}}. \end{array} $$

The test statistic *T*^*B*^ is approximately standard normal distributed. Thus, the hypothesis of equal weighted survival probabilities between the groups is rejected if *T*^*R*^≤−*z*_1−*α*_, where *z*_1−*α*_ is the (1−*α*)-quantile of the standard normal distribution and *α* is the one-sided significance level.

### Simulation study

To provide a systematic comparison of the methods described in the previous section, we conducted a simulation study. As before, we consider a composite endpoint combining two event types; one fatal event given by death (*D*) and one non-fatal given by myocardial infarction (*M*). For all scenarios 200 individuals per data set were generated with 100 in each treatment group. A follow-up of three years was assumed, i.e. adminstrative censoring for an individual follow-up after three years. Hence, the maximum number of events is limited by this observational period and impacted by the underlying event distribution. The mean event count per scenario is given in Table 3. In the simulation, we additionally limited the maximal event count per individual to 100. Patients who do not have an event up to that time point remain in the analysis with a censored time point. The effect estimates and tests will be evaluated at three years, i.e. at the end of the study period.

In Table 2 the simulation scenarios are listed. Columns 2 to 5 show the assumed underlying hazard functions. The hazards are displayed as products of the baseline hazards and the cause-specific effects to underline the assumption of equal baseline hazards. The cause-specific hazard are assumed to be either exponentially or Weibull distributed. The continuous event times are generated as described by Bender et al. [23] for the fatal event and as described by Jahn-Eimermacher et al. [24] for the non-fatal recurrent event. To gain first insights into the performance of the three methods we consider scenarios where the baseline hazards and hazard ratios do not change dependent on previous events, i.e. there are also no strata-specific effects.

**Table 2:** Simulation scenarios

Scen.	$\lambda _{M,j}^{I}(t)$	$\lambda _{D,j}^{I}(t)$	$\lambda _{M,j}^{C}(t)$	$\lambda _{D,j}^{C}(t)$	Weight for fatal event		Weight for non-fatal event	
					Wei-Lachin	Rauch and Bakal	Wei-Lachin	Rauch and Bakal
1*a*	0.25·0.5	0.25·0.5	0.25	0.25	0.5263	1	0.4737	0.9
1*b*	proportional time independent hazards for event type				0.5882	1	0.4118	0.7
1*c*	assumptions for approaches by Wei-Lachin and Rauch fulfilled				0.6667	1	0.3333	0.5
1*d*					0.7692	1	0.2308	0.3
1*e*					0.9091	1	0.0909	0.1
2*a*	0.25·0.5	0.25·0.7	0.25	0.25	0.5263	1	0.4737	0.9
2*b*	see Scenario 1 for underlying assumptions				0.5882	1	0.4118	0.7
2*c*					0.6667	1	0.3333	0.5
2*d*					0.7692	1	0.2308	0.3
2*e*					0.9091	1	0.0909	0.1
3*a*	0.25·0.7	0.25·0.5	0.25	0.25	0.5263	1	0.4737	0.9
3*b*	see Scenario 1 for underlying assumptions				0.5882	1	0.4118	0.7
3*c*					0.6667	1	0.3333	0.5
3*d*					0.7692	1	0.2308	0.3
3*e*					0.9091	1	0.0909	0.1
4*a*	0.25·1.5	0.25·0.7	0.25	0.25	0.5263	1	0.4737	0.9
4*b*	see Scenario 1 for underlying assumptions				0.5882	1	0.4118	0.7
4*c*					0.6667	1	0.3333	0.5
4*d*					0.7692	1	0.2308	0.3
4*e*					0.9091	1	0.0909	0.1
5*a*	0.25·0.7	0.25·1.5	0.25	0.25	0.5263	1	0.4737	0.9
5*b*	see Scenario 1 for underlying assumptions				0.5882	1	0.4118	0.7
5*c*					0.6667	1	0.3333	0.5
5*d*					0.7692	1	0.2308	0.3
5*e*					0.9091	1	0.0909	0.1
6*a*	0.25·0.5	0.1*t*·0.7	0.25	0.1*t*	0.5263	1	0.4737	0.9
6*b*	proportional hazards for each event type				0.5882	1	0.4118	0.7
6*c*	time dependent hazards for one event type				0.6667	1	0.3333	0.5
6*d*	assumptions for approaches by Wei-Lachin and Rauch fulfilled				0.7692	1	0.2308	0.3
6*e*					0.9091	1	0.0909	0.1
7*a*	0.25·0.7	0.1*t*·0.5	0.25	0.1*t*	0.5263	1	0.4737	0.9
7*b*	see Scenario 6 for underlying assumptions				0.5882	1	0.4118	0.7
7*c*					0.6667	1	0.3333	0.5
7*d*					0.7692	1	0.2308	0.3
7*e*					0.9091	1	0.0909	0.1
8*a*	0.1*t*·0.5	0.25·0.7	0.1*t*	0.25	0.5263	1	0.4737	0.9
8*b*	see Scenario 6 for underlying assumptions				0.5882	1	0.4118	0.7
8*c*					0.6667	1	0.3333	0.5
8*d*					0.7692	1	0.2308	0.3
8*e*					0.9091	1	0.0909	0.1
9*a*	0.1*t*·0.7	0.25·0.5	0.1*t*	0.25	0.5263	1	0.4737	0.9
9*b*	see Scenario 6 for underlying assumptions				0.5882	1	0.4118	0.7
9*c*					0.6667	1	0.3333	0.5
9*d*					0.7692	1	0.2308	0.3
9*e*					0.9091	1	0.0909	0.1
10*a*	0.192*t*^−0.2^	0.084*t*^−0.3^	0.28*t*^−0.3^	0.32*t*^−0.2^	0.5263	1	0.4737	0.9
10*b*	non-proportional hazards for both event types (Weibull distributions)				0.5882	1	0.4118	0.7
10*c*	assumptions for approach by Wei-Lachin not fulfilled				0.6667	1	0.3333	0.5
10*d*	assumptions for approach by Rauch et al. fulfilled				0.7692	1	0.2308	0.3
10*e*					0.9091	1	0.0909	0.1
11*a*	0.084*t*^−0.3^	0.192*t*^−0.2^	0.32*t*^−0.2^	0.28*t*^−0.3^	0.5263	1	0.4737	0.9
11*b*	see Scenario 10 for underlying assumptions				0.5882	1	0.4118	0.7
11*c*					0.6667	1	0.3333	0.5
11*d*					0.7692	1	0.2308	0.3
11*e*					0.9091	1	0.0909	0.1

Scen. =Scenario; $\lambda _{M,j}^{I}(t), \lambda _{D,j}^{I}(t), \lambda _{M,j}^{C}(t)$, and $\lambda _{D,j}^{C}(t)$ are the hazards for the non-fatal (*M*) and fatal event (*D*) in the intervention (*I*) and control (*C*) group for all strata *j*, respectively

The considered weights for the different weighting approaches are listed in columns 6 to 9. For the Wei-Lachin approach the weights for the fatal and non-fatal event are chosen to sum up to 1 and such that the ratio between the weights is equal to the weight ratio of the other two approaches $\frac {w^{*}_{M}}{w^{*}_{D}}$ as given in Column 9. For the fatal event the weight is set to 1 for the approach by Rauch et al. and Bakal et al.. The weights for the non-fatal event are ranging between 0.1 and 0.9 for the approach by Rauch et al. and Bakal et al.. Scenarios *a*−*e* depict those weight changes.

For Scenario 1 equal time independent baseline hazards for the event types are assumed as well as equal cause-specific effects. In Scenario 2 to 5 different cause-specific effects are assumed. In the Scenarios 4 and 5 the cause-specific effects of the two event types point into opposite directions. In the Scenarios 6 to 9 one baseline hazard is time dependent but the cause-specific effects and weights are as for the Scenarios 2 and 3. For Scenarios 10 and 11 non-proportional cause-specific hazards are considered, resulting in a time dependent effect estimand.

For each scenario 2000 data sets were simulated and analyzed. In case of non-convergence for an approach the data set will be excluded.

We used the statistic software R (Version 3.6.1 and 4.0.3) [20] for the simulation study. R uses the Mersenne twister [25] for generating random numbers.

### Example data

To illustrate the methods further we apply all three methods to an open source clinical study data set available within the R package frailtypack [26] named *readmission*. This data is taken from a study published by Gonzales et al. in 2005 [27]. They analyzed 403 patients with a new diagnosis of colorectal cancer who had a surgery between January 1996 and December 1998. They were actively followed up until 2002. Time to rehospitalization and time to death after surgery were included in the dataset. A total of 458 readmssions were observed and 112 patients died within the study period. The maximal event count for a patient in the data set is 23 and the mean individual event count is 2.6 (± 2.8). The primary study aim is to compare the number of observed fatal and non-fatal events between patients who received chemotherapy (217 (53.8%)) and those who did not (186 (46.2%)). Since the event death as a fatal event is assumed to be more clinical relevant a higher weight will be assigned to death as compared to readmission. However, results of different weighting schemes will be shown for illustration. In clinical practice and confirmatory trials the weighting scheme should be pre-specified and other weighting schemes as well as the unweighted case can be chosen as sensitivity analysis.

## Results

### Results of simulation study

In Table 3 the results of the simulation study are displayed.

We start by looking at the estimands, estimator, and corresponding root mean squared error for the Wei-Lachin approach and the approach by Rauch et al. since the deviation from the true simulated values is of primary interest. Recall that for the approach by Bakal et al. there is no estimand and thus no estimator.

The true effects (estimands) for the Wei-Lachin approach and the approach by Rauch et al. are in most scenarios similar in magnitude and even equal in some cases (if cause-specific hazards and hazard ratios are equal between event types). With less influence of the recurrent event (i.e. a smaller weight; going from scenario a to e) the composite effect gets closer to the effect of the terminal event that is the effect of the terminal event tends to suppress the effect of the recurrent non-fatal event. This effect is more or less prominent depending on the underlying cause-specific hazards.

**Table 3:** Simulation results

Scen.	Mean amount of events (sd)			Power			Estimand		Estimator		$\sqrt {MSE}^{**}$	
							at FU=3		(sd) ^∗^			
	Non	Fatal	Per	Wei-	Rauch	Bakal	Wei-	Rauch	Wei-	Rauch	Wei-	Rauch
	fatal		individual	Lachin			Lachin		Lachin		Lachin	
1*a*	84.05 (9.66)	84.14 (6.71)	3.37 (0.70)	0.99	1.00	0.98	0.5	0.5	0.50 (1.18)	0.50 (1.18)	0.17	0.17
1*b*				0.99	1.00	0.98	0.5	0.5	0.50 (1.18)	0.50 (1.18)	0.17	0.17
1*c*				0.99	1.00	0.97	0.5	0.5	0.50 (1.19)	0.50 (1.19)	0.17	0.17
1*d*				0.98	0.99	0.95	0.5	0.5	0.50 (1.20)	0.50 (1.20)	0.19	0.19
1*e*				0.93	0.98	0.92	0.5	0.5	0.50 (1.23)	0.50 (1.23)	0.21	0.21
2*a*	82.01 (9.58)	93.69 (6.88)	3.36 (0.70)	0.92	0.97	0.72	0.60	0.61	0.59 (1.17)	0.61 (1.18)	0.16	0.16
2*b*				0.89	0.95	0.70	0.61	0.62	0.60 (1.18)	0.62 (1.18)	0.16	0.17
2*c*				0.84	0.92	0.66	0.63	0.63	0.62 (1.18)	0.63 (1.19)	0.16	0.17
2*d*				0.72	0.86	0.59	0.65	0.65	0.64 (1.19)	0.65 (1.20)	0.17	0.18
2*e*				0.51	0.71	0.49	0.68	0.68	0.68 (1.22)	0.68 (1.22)	0.20	0.20
3*a*	96.60 (10.43)	84.14 (6.71)	3.47 (0.69)	0.94	0.98	0.92	0.59	0.60	0.58 (1.17)	0.59 (1.17)	0.16	0.16
3*b*				0.94	0.99	0.93	0.57	0.58	0.57 (1.17)	0.58 (1.18)	0.16	0.16
3*c*				0.95	0.99	0.93	0.56	0.57	0.56 (1.18)	0.56 (1.18)	0.17	0.17
3*d*				0.94	0.98	0.92	0.54	0.55	0.54 (1.20)	0.54 (1.20)	0.18	0.18
3*e*				0.91	0.97	0.90	0.52	0.52	0.51 (1.23)	0.52 (1.23)	0.21	0.20
4*a*	140.43 (12.97)	93.69 (6.88)	4.48 (0.81)	0.02	0.03	0.04	1.00	1.08	1.00 (1.15)	1.07 (1.16)	0.14	0.15
4*b*				0.05	0.06	0.04	0.96	1.03	0.96 (1.16)	1.03 (1.17)	0.15	0.15
4*c*				0.10	0.12	0.07	0.90	0.97	0.90 (1.17)	0.97 (1.17)	0.16	0.16
4*d*				0.19	0.26	0.12	0.83	0.89	0.83 (1.19)	0.89 (1.18)	0.17	0.17
4*e*				0.32	0.46	0.25	0.75	0.77	0.75 (1.21)	0.77 (1.21)	0.19	0.19
5*a*	84.38 (9.90)	120.49 (6.66)	3.42 (0.71)	0.01	0.02	0.00	1.05	1.12	1.04 (1.16)	1.13 (1.17)	0.15	0.16
5*b*				0.01	0.01	0.00	1.10	1.17	1.09 (1.16)	1.18 (1.17)	0.15	0.16
5*c*				0.00	0.01	0.00	1.16	1.23	1.16 (1.16)	1.24 (1.18)	0.15	0.16
5*d*				0.00	0.00	0.00	1.26	1.32	1.26 (1.17)	1.32 (1.18)	0.15	0.17
5*e*				0.00	0.00	0.00	1.40	1.43	1.40 (1.19)	1.43 (1.19)	0.17	0.18
6*a*	99.16 (10.16)	63.25 (6.44)	3.52 (0.71)	0.87	0.98	0.71	0.60	0.61	0.59 (1.19)	0.59 (1.19)	0.18	0.18
6*b*				0.81	0.96	0.68	0.61	0.63	0.60 (1.20)	0.60 (1.20)	0.18	0.19
6*c*				0.72	0.94	0.62	0.63	0.64	0.62 (1.21)	0.61 (1.21)	0.19	0.20
6*d*				0.56	0.85	0.53	0.65	0.66	0.64 (1.23)	0.64 (1.23)	0.21	0.21
6*e*				0.36	0.64	0.38	0.68	0.69	0.67 (1.27)	0.67 (1.28)	0.24	0.24
7*a*	114.14 (11.00)	56.32 (6.14)	3.62 (0.72)	0.88	0.95	0.82	0.59	0.59	0.58 (1.20)	0.61 (1.18)	0.18	0.17
7*b*				0.87	0.96	0.82	0.57	0.57	0.57 (1.21)	0.60 (1.19)	0.19	0.18
7*c*				0.85	0.96	0.82	0.56	0.56	0.55 (1.23)	0.58 (1.20)	0.21	0.19
7*d*				0.82	0.95	0.81	0.54	0.54	0.53 (1.26)	0.56 (1.22)	0.23	0.20
7*e*				0.75	0.91	0.76	0.52	0.52	0.51 (1.30)	0.52 (1.28)	0.27	0.24
8*a*	39.86 (6.01)	93.69 (6.88)	1.97 (0.39)	0.78	0.88	0.47	0.60	0.60	0.59 (1.22)	0.64 (1.19)	0.20	0.19
8*b*				0.78	0.85	0.46	0.61	0.61	0.60 (1.21)	0.65 (1.19)	0.19	0.19
8*c*				0.76	0.81	0.46	0.63	0.63	0.62 (1.20)	0.66 (1.20)	0.18	0.19
8*d*				0.69	0.75	0.44	0.65	0.65	0.64 (1.20)	0.67 (1.22)	0.18	0.19
8*e*				0.52	0.67	0.42	0.68	0.68	0.67 (1.21)	0.69 (1.22)	0.19	0.20
9*a*	47.27 (6.58)	84.14 (6.71)	2.02 (0.35)	0.49	0.97	0.90	0.59	0.60	0.58 (1.21)	0.57 (1.19)	0.19	0.18
9*b*				0.88	0.97	0.90	0.57	0.59	0.57 (1.20)	0.56 (1.19)	0.18	0.19
9*c*				0.91	0.98	0.90	0.56	0.58	0.56 (1.20)	0.54 (1.20)	0.18	0.19
9*d*				0.92	0.98	0.90	0.54	0.55	0.54 (1.21)	0.53 (1.21)	0.19	0.20
9*e*				0.90	0.97	0.89	0.52	0.52	0.51 (1.23)	0.51 (1.24)	0.21	0.22
10*a*	111.94 (11.53)	84.44 (6.64)	4.07 (0.85)	1.00	1.00	1.00	0.41	0.45	0.41 (1.18)	0.44 (1.17)	0.17	0.16
10*b*				1.00	1.00	1.00	0.38	0.42	0.39 (1.19)	0.42 (1.17)	0.18	0.16
10*c*				1.00	1.00	1.00	0.35	0.38	0.36 (1.21)	0.39 (1.18)	0.19	0.16
10*d*				1.00	1.00	1.00	0.31	0.34	0.33 (1.22)	0.35 (1.19)	0.22	0.18
10*e*				1.00	1.00	1.00	0.27	0.27	0.29 (1.27)	0.30 (1.24)	0.26	0.23
11*a*	85.59 (10.21)	101.60 (7.06)	4.06 (0.88)	1.00	1.00	0.96	0.44	0.48	0.43 (1.19)	0.46 (1.18)	0.17	0.17
11*b*				1.00	1.00	0.95	0.47	0.52	0.46 (1.19)	0.49 (1.18)	0.17	0.17
11*c*				1.00	1.00	0.92	0.52	0.56	0.49 (1.19)	0.52 (1.18)	0.18	0.18
11*d*				0.95	0.97	0.86	0.58	0.62	0.53 (1.19)	0.56 (1.19)	0.20	0.20
11*e*				0.76	0.87	0.72	0.69	0.71	0.60 (1.21)	0.63 (1.21)	0.24	0.23

Scen. =Scenario; sd=standard deviation; FU=follow-up; ^∗^at FU=3 with sd based on logarithmic scale and afterwards back-transformed; ^∗∗^ root of mean squared error (MSE) based on the logarithmized effect at FU=3

The estimators and corresponding standard deviations, and thus the mean squared errors, are also similar (or equal) for the two approaches within all scenarios. The estimators also depict that with less influence of the recurrent event the composite effect gets closer to the effect of the terminal event.

For the approaches by Wei-Lachin and by Rauch et al. it is seen that with the decreasing weight for the recurrent event the variability in the estimator increases (i.e. higher mean squared error is observed when changing from Scenarios a to e). The mean squared error is highest (mostly due to higher variability in estimation) in scenarios with time dependent hazards (Scenarios 6 to 11). The root mean squared error is best to compare the bias and variability of the estimators. Since they are mostly almost the same between the methods, the Wei-Lachin approach and approach by Rauch et al. perform equally well in terms of mean squared error.

For the Scenarios 10 and 11, the composite effect is time dependent but in our Scenarios we only evaluate and test the effect at a given time point, i.e. three years. In this case the estimated effect might be closer to the true underlying effect at some time points but at other time points estimation might result in major bias. In Scenario 5 a composite estimand greater than 1, i.e. effect in favor of the control, is given. The estimators capture this. Since we consider a one-sided null-hypothesis the power observed within Scenario 5 is almost 0. In Scenario 4 the composite estimand is closer to 1 than in other scenarios (except Scenario 5). Hence, smaller power values are observed due to the one-sided study design.

The following observatios are made for the power values: The power for the approach by Bakal et al. is the lowest in most scenarios. In some scenarios the power for the approach by Bakal et al. is similar to the power observed within the Wei-Lachin approach. For the approach by Rauch et al. the highest power is seen in most scenarios. For Scenario 1a-e where the estimand remains the same for all weighting schemes it is seen that the power decreases with decreasing weight for the non-fatal event (i.e. from Scenario 1a to 1e). In Scenarios 3 and 7 the power decreases although the estimands increase. In these scenarios a smaller effect for the recurrent event is assumed and while decreasing the weight its influence on the effect estimate decreases as well and hence the power is based on the less occurring fatal event which leads to more variability. In scenarios where the composite effect approaches 1 with a smaller weight for the recurrent event (i.e Scenarios 2 and 6) the power decreases radically.

### Results of application

Table 4 shows the results for the example dataset. For different weighting schemes the p-values are given for a one-sided test for all three approaches. For the method by Wei and Lachin and Rauch et al. the result for the estimated weighted effect measure is shown.

**Table 4:** Application results

Weighting scheme	p-value ^∗^			Estimator ^∗∗^	
	Wei-Lachin	Rauch	Bakal	Wei-Lachin	Rauch
Weight for death: 1	0.34	0.10	0.40	1.05	1.38
Weight for readmission: 1					
Weight for death: 1	0.29	0.09	0.39	1.07	1.39
Weight for readmission: 0.9					
Weight for death: 1	0.21	0.07	0.37	1.11	1.40
Weight for readmission: 0.7					
Weight for death: 1	0.14	0.05	0.32	1.17	1.42
Weight for readmission: 0.5					
Weight for death: 1	0.09	0.04	0.23	1.25	1.43
Weight for readmission: 0.3					
Weight for death: 1	0.05	0.03	0.05	1.36	1.44
Weight for readmission: 0.1					

^∗^one-sided test in favour of no chemotherapy; ^∗∗^ estimated weighted composite effect at end of study period for chemotherapy vs. no chemotherapy; composite endpoint = (recurrent) hospitalization plus death

The estimated unweighted cause-specific hazard ratios comparing patients with chemotherapy to patients without chemotherapy are 0.77 for the event readmission and 1.44 for the event death. Note, that they point into opposite directions, i.e. patients who received chemotherapy have a higher chance to die compared to patients who did not receive chemotherapy. In contrast, the patients who are treated with chemotherapy have a lower chance to experience readmission compared to those with chemotherapy. This can also be seen in the results of all three methods since with a lower weight for hospitalization the difference between the patients with chemotherpy and those without increases, i.e. depict more and more the difference seen for the death event alone as seen in the estimator which becomes larger. In the example, the difference between the estimated weighted effect measure for the approach by Wei and Lachin and Rauch et al. is more prominent than in the simulation study which might be due to the higher event count for the non-fatal event. The p-value within the approach by Bakal et al. is always the highest and hence shows only a significance if readmission is ingored, i.e. has a really low weight, in the analysis.

## Discussion

The analysis of composite endpoints combining events of different clinical relevance with potentially recurrent events is a challenging task in cardiovascular or oncologic trials. Therefore, we are the first to compare three methods that were proposed in the literature to give an overview of their properties in different clinical data situations. This should help the applied researcher to choose an adequate method in future clinical trials. The proposed methods differ in their properties and assumptions. However, for all approaches the choice of the weighting scheme should be based on clinical relevance of event types.

Wei and Lachin proposed an approach where the pre-specified relative weights work on the cause-specific log-hazard ratios. For this approach not only an estimand is given but also a closed formula for a corresponding variance and thus confidence intervals. The power within this approach gained via the multivariate testing procedure was mostly between the power of the other two approaches in our simulation study but more similar to those gained for the approach by Bakal et al.. This can be explained by the fact the weights work on the cause-specific effects, which are thus estimated separately. The combined effect is then a weighted average of the individually estimated cause-specific effects. The estimation is thus based on a smaller event count which results in a higher variability for each cause-specific effect, i.e. higher variances are combined in the multivariate procedure. Furthermore, because the weights work only on the cause-specific effects the event count and distribution of events is not considered. Thus, a high cause-specific effect which is based on a low event number has still a great impact on the weighted composite effect which might be questionable as an effect based on a small event count has a high standard error. On the other hand, also an effect estimated based on high uncertainty can be relevant for clinical practice, so there are several views on this aspect.

Rauch et al. proposed an approach that extends the common all-cause hazard ratio and thereby naturally proposed an underlying estimand. Although an estimand is given, no closed formula for a corresponding variance and thus no confidence intervals could be derived. However, the corresponding weight based log-rank test (which was extended to a stratified approach in the present study to account for recurrent events) showed the highest power in our simulation study with similar properties (e.g. mean squared error) as compared to the approach by Wei and Lachin. Pre-specified relevance weights work on the cause-specific hazards and thus on the event count. Hence, the weighted all-cause effect does not exclusively rely on the cause-specific effects. This is an advantage because in a situation where a low event number goes along with an observed high cause-specific effect, the influence on the weighted composite effect is reduced, i.e. a more reliable effect estimate can be gained.

Bakal et al. proposed a weighted estimate for survival probabilities in a Kaplan-Meier type estimation approach. They did not provide an estimand and thus no effect estimator can be reasonably reported. Pre-defined relevance weights within this approach work on the event count as well as on the number of patients at risk. Although, the principle concept of Bakal’s approach seems appealing, the methods lacks a theoretical foundation, an underlying model and a prespecified estimand. Our results moreover show the lowest power for this approach in most scenarios. We therefore cannot recommend to use the approach by Bakal et al..

For the approaches by Wei and Lachin and Rauch et al. however, the results should be interpreted with care if the proportional hazards assumption is not met for the components. In this case the composite effect is time dependent which is not captured whithin these approaches, i.e. they assume constant effects. Hence the estimated effect might be correctly estimated at some time points but at others major bias might be observed. For the non-parametric approach by Bakal et al. there is no assumption about proportional hazards but since they did not state a theoretical model it is not possible to evaluate the performance in terms of bias.

The Wei-Lachin approach assumes a constant composite effect over time. Within the approach by Bakal et al. time dependence is also not considered. Although for the approach by Rauch et al. a time dependent estimate can be gained the stratified weight based log-rank test does not incorporate this time dependence.

This means, that the approach by Wei et al. as well as the approach by Rauch et al. make strong assumptions. Proportional hazards are needed for the different causes and on strata level which is usually not met in clinical practice. Rauch et al. developed their estimand based on the assumption of a specific underlying survival distribution (parametric model). To derive a non-parametric formulation equal cause-specific baseline hazards are needed. However, it was shown that this non-parametric approach is robust against a miss-classification [15].

Furthermore, a disadvantage of all three methods is that the dependence between the fatal event and the recurrent event process is not modeled, which could be addressed by joint frailty models [8], [9].

In future studies the evaluation of the illustrated methods within a two-sided test problem might be of interest to confirm our results for the one-sided case (we do not assume that there will be any differences). Furthermore, the evaluation of the type one error in different scenarios should be evaluated since this was only marginally captured within this work, i.e. only once when the weighted composite estimand was 1 in the Wei-Lachin approach (Scenario 4a). Thereby, it should be noted that there are several constellations which yield a weighted estimand of 1. Robust standard errors should mostly be applied within recurrent time to event analysis, which might also influence statistical significance and type one error and hence it should be evaluated how they can be incorporated within a log-rank type test statistic, since the log-rank type test statistics (Rauch et al., Bakal et al.) do not allow such an extension at the moment. More complex scenarios should also be evaluated, i.e. where a correlation between event types is simulated or where more than two event types are considered. We considered only the three methods evaluated in this work where it was originally described that for the weighted components within a composite endpoint an extension to multiple events per patient is possible. However, it still might be useful to compare other methods for weighted composite endpoints, e.g. by Buyse [10]. Buyse described how to perform generalized pairwise comparisons between two groups of observations with prioritized outcome. As this approach is not based on a time to event model, we neglected it within this paper.

We were only interested in the estimation of the composite effect, but in clinical studies the cause-specific effects should also be reported as recommended by several guidelines [28–30]. It should also be noted that the events considered in the composite endpoint should all be harmful or all be favorable, a mixture of harmful and favorable events must be avoided.

## Conclusion

In conclusion, for clinical studies where a two groups comparison with respect to a composite endpoint combining (recurrent) events of different clinical relevance is of interest two approaches might be recommended which have different pros and cons: The approach by Rauch et al. can be recommended due to its intuitive interpretation although it provides only bootstrap confidence intervals for the effect estimate. The approach by Wei and Lachin might be preferred, when all event types show a reasonable event count and when the derivation of confidence intervals is central. The approach by Bakal et al. in its current form should be applied with care as a theoretical foundation is lacking.

## Supplementary Information

**Additional file 1** Additional file for: ’Weighted composite time to event endpoints with recurrent events: comparison of three analytical approaches’.

## Data Availability

The datasets used and/or analysed during the current study available from the corresponding author on reasonable request.

## Abbreviations

B: Bakal
C: Control
D: Death
I: Intervention
M: Myocardial infarction
R: Rauch
L: Wei-Lachin
w: weight
