# Supplementary material for: Weighted composite time to event endpoints with recurrent events: comparison of three analytical approaches
Source: BMC Med Res Methodol. 2022 Feb 5;22:38. doi: 10.1186/s12874-022-01511-1 (PMC8818231; doi:10.1186/s12874-022-01511-1)
Supplement: Supplementary file 1 — Additional file 1 Additional file for: ’Weighted composite time to event endpoints with recurrent events: comparison of three analytical approaches’. [file 12874_2022_1511_MOESM1_ESM.pdf]

RESEARCH

Additional file for: 'Weighted Composite Time To Event Endpoints with Recurrent Events: Comparison of Three Analytical Approaches'

Ann-Kathrin Ozga and Geraldine Rauch

Full list of author information is available at the end of the article

1 Stratified weight-based log-rank test

The test-statistic for the approach by Rauch et al. (see Section *Approach by Rauch et al.* of main text) is given as follows:

$$T^R = \frac{\sum_{j=1}^{J}\sum_{k=1}^{K}\left[\left(w_D^R e_{D,jk}^I + w_M^R e_{M,jk}^I\right) - \frac{n_{jk}^I}{n_{jk}^I + n_{jk}^C}\left(w_D^R e_{D,jk} + w_M^R e_{M,jk}\right)\right]}{\sqrt{\sum_{j=1}^{J}\sum_{k=1}^{K}\frac{n_{jk}^I n_{jk}^C\left[\left(n_{jk}^I + n_{jk}^C\right)\left[\left(w_D^R\right)^2 e_{D,jk} + \left(w_M^R\right)^2 e_{M,jk}\right] - \left(w_D^R\right)^2 e_{D,jk}^2 - \left(w_M^R\right)^2 e_{M,jk}^2\right]}{\left(n_{jk}^I + n_{jk}^C\right)^2\left(n_{jk}^I + n_{jk}^C - 1\right)}}}$$

- $j = 1, ..., J$ are the strata, i.e. all first events (death D or myocardial infarction M) are in the first stratum, all second events (death D or myocardial infarction M) are in the second stratum, and so on.
- K_j are the number of event times in stratum j, i.e. $k = 1, ..., K$ indicate the number of events within a stratum.
- w_D^R and w_M^R are the weights for death and myocardial infarction, respectively.
- $e_{D,jk}^I$ are the number of death-events in stratum j at event time point t_{jk} within the intervention group I.
- $e_{M,jk}^I$ are the number of myocardial infarction-events in stratum j at event time point t_{jk} within the intervention group I.
- $e_{D,jk}$ are the number of death-events in stratum j at event time point t_{jk} (for intervention I and control C combined).
- $e_{M,jk}$ are the number of myocardial infarction-events in stratum j at event time point t_{jk} (for intervention I and control C combined).
- n_{jk}^I are the number of individuals at risk in stratum j at event time t_{jk} for the intervention group.
- n_{jk}^C are the number of individuals at risk in stratum j at event time t_{jk} for the control group.

2 Example Bakal et al.

Table 1 Example data set to illustrate approach by Bakal et al.

ID	time	weight
1	1	0.3
1	2	0.3
1	3	0.3
1	4	1
2	1	0.3
2	4	1
3	5	0.3
3	6	0.3
3	7	1

- First step:
  - Calculate individual score at all (whole dataset) distinct ordered event time points ($s_i(t_k)$) with $i = 1, ...n$, K_j as above, $s_i(0) = 1$, and $t_0 = 0$:

    $$s_i(t_k) = s_i(t_{k-1}) - \Big[s_i(t_{k-1}) \cdot w_i^B(t_k)\Big]$$

  - **In example:** n=3, K=7

    Individual $ID = 1$:

    at $t_1 = 1$ the score is: $s_1(1) = s_1(t_0) - [\ s_1(t_0) \cdot w_1^B(1)] = 1 - (1 \cdot 0.3) = 0.7$

    at $t_2 = 2$ the score is: $s_1(2) = s_1(t_1) - [s_1(t_1) \cdot w_1^B(2)] = 0.7 - (0.7 \cdot 0.3) = 0.49$

    at $t_3 = 3$ the score is: $s_1(3) = s_1(t_2) - [s_1(t_2) \cdot w_1^B(3)] = 0.49 - (0.49 \cdot 0.3) = 0.343$

    at $t_4 = 4$ the score is: $s_1(4) = s_1(t_3) - [s_1(t_3) \cdot w_1^B(4)] = 0.343 - (0.343 \cdot 1) = 0$

    at $t_5 = 5$ the score is: $s_1(5) = 0$

    at $t_6 = 6$ the score is: $s_1(6) = 0$

    at $t_7 = 7$ the score is: $s_1(7) = 0$

    Individual $ID = 2$:

    at $t_1 = 1$ the score is: $s_2(1) = s_2(t_0) - [s_2(t_0) \cdot w_2^B(1)] = 1 - (1 \cdot 0.3) = 0.7$

    at $t_2 = 2$ the score is: $s_2(2) = s_2(t_1) - [s_2(t_1) \cdot w_2^B(2)] = 0.7 - (0.7 \cdot 0) = 0.7$

    at $t_3 = 3$ the score is: $s_2(3) = s_2(t_2) - [s_2(t_2) \cdot w_2^B(3)] = 0.7 - (0.7 \cdot 0) = 0.7$

    at $t_4 = 4$ the score is: $s_2(4) = s_2(t_1) - [s_2(t_1) \cdot w_2^B(4)] = 0.7 - (0.7 \cdot 1) = 0$

    at $t_5 = 5$ the score is: $s_2(5) = 0$

    at $t_6 = 6$ the score is: $s_2(6) = 0$

    at $t_7 = 7$ the score is: $s_2(7) = 0$

Individual $ID = 3$:

at $t_1 = 1$ the score is: $s_1(1) = s_3(t_0) - [s_3(t_0) \cdot w_3^B(1)] = 1 - (1 \cdot 0) = 1$

at $t_2 = 2$ the score is: $s_1(2) = s_3(t_1) - [s_3(t_1) \cdot w_3^B(2)] = 1 - (1 \cdot 0) = 1$

at $t_3 = 3$ the score is: $s_1(3) = s_3(t_2) - [s_3(t_2) \cdot w_3^B(3)] = 1 - (1 \cdot 0) = 1$

at $t_4 = 4$ the score is: $s_1(4) = s_3(t_3) - [s_3(t_3) \cdot w_3^B(4)] = 1 - (1 \cdot 0) = 1$

at $t_5 = 5$ the score is: $s_1(5) = s_3(t_4) - [s_3(t_4) \cdot w_3^B(5)] = 1 - (1 \cdot 0.3) = 0.7$

at $t_6 = 6$ the score is: $s_1(6) = s_3(t_5) - [s_3(t_5) \cdot w_3^B(6)] = 0.7 - (0.7 \cdot 0.3) = 0.49$

at $t_7 = 7$ the score is: $s_1(7) = s_3(t_6) - [s_3(t_6) \cdot w_3^B(7)] = 0.49 - (0.49 \cdot 1) = 0$

- Second step:
  - Calculate weighted survival probabilities

    $$KM^B(t_k) = KM^B(t_{k-1}) \cdot \left(1 - \frac{\sum_{1=1}^n s_i(t_{k-1}) - \sum_{i=1}^n s_i(t_k)}{\sum_{i=1}^n s_i(t_{k-1})}\right)$$

    With $KM^B(t_0) = 1$

  - **In example:**

    $$KM^B(t_1) = 1 \cdot (1 - \frac{3-2.4}{3}) = 0.8$$

    $$KM^B(t_2) = 0.8 \cdot (1 - \frac{2.4-2.19}{2.4}) = 0.73$$

    $$KM^B(t_3) = 0.73 \cdot (1 - \frac{2.19-2.043}{2.19}) = 0.681$$

    $$KM^B(t_4) = 0.681 \cdot (1 - \frac{2.043-1}{2.043}) = 0.33$$

    $$KM^B(t_5) = 0.33 \cdot (1 - \frac{1-0.7}{1}) = 0.23$$

    $$KM^B(t_6) = 0.23 \cdot (1 - \frac{0.7-0.49}{0.7}) = 0.209$$

    $$KM^B(t_7) = 0.209 \cdot (1 - \frac{0.49}{0.49}) = 0$$

3 Weight calculation for the weights in the Wei-Lachin approach

To gain the same weight relation in the Wei-Lachin approach as for the weights in the weighted hazards approach or Bakal's approach the following calculation have to be conducted under the assumption that $w_D^B = 1$ and the relation is denoted by $\frac{w_M^B}{w_D^B}$ with D as the fatal event and M as the non-fatal event:

For weighted hazards weights or Bakal's weights:

$\frac{w_M^B}{w_D^B} = x \rightarrow w_M^B = x$

For Wei-Lachin weights:

$\frac{w_M^L}{w_D^L} = x$ and $w_M^L + w_D^L = 1$

$w_M^L = xw_D^L$ and $w_M^L = 1 - w_D^L$

$xw_D^L \overset{!}{=} 1 - w_D^L \rightarrow w_D^L = \frac{1}{1+x}$ and $w_M^L = 1 - \frac{1}{1+x}$
